# Carotid Calcification in Mice: A New Model to Study the Effects of Arterial Stiffness on the Brain

**DOI:** 10.1161/JAHA.113.000224

**Published:** 2013-06-21

**Authors:** Nataliya Sadekova, Diane Vallerand, Edgar Guevara, Frédéric Lesage, Hélène Girouard

**Affiliations:** 1 Department of Pharmacology, Faculty of Medicine, Université de Montréal, Montreal, Quebec, Canada (N.S., D.V., H.G.); 2 Research Center, Montreal Heart Institute, 5000 Bélanger Est, Montreal, Quebec, Canada (E.G., F.L.)

**Keywords:** arterial stiffness, brain, calcium chloride, carotid

## Abstract

**Background:**

Arterial stiffness has been identified as an important risk factor for cognitive decline. However, its effects on the brain's health are unknown, and there is no animal model available to study the precise impact of arterial stiffness on the brain. Therefore, the objective of the study was to develop and characterize a new model specific to arterial stiffness in order to study its effects on the brain.

**Methods and Results:**

Calcium chloride (CaCl_2_) was applied to carotid arteries of mice, inducing an increase in collagen distribution and intima–media thickness, a fragmentation of elastin, a decrease in arterial compliance and distensibility, and an increase in cerebral blood flow pulsatility (n=3 to 11). Calcium deposits were only present at the site of CaCl_2_ application, and there was no increase in systemic blood pressure or change in vessel radius making this model specific for arterial stiffness. The effects of carotid stiffness were then assessed in the brain. Carotid calcification induced an increase in the production of cerebral superoxide anion and neurodegeneration, detected with Fluoro‐Jade B staining, in the hippocampus (n=3 to 5), a key region for memory and cognition.

**Conclusions:**

A new model of arterial stiffness based on carotid calcification was developed and characterized. This new model meets all the characteristics of arterial stiffness, and its specificity allows the study of the effects of arterial stiffness on the brain.

## Introduction

Arterial stiffness is an important risk factor and a predictor of cognitive impairment and dementia in the elderly.^1–3^ It has been shown that central arterial stiffness, evaluated by pulse‐wave velocity between carotid and femoral arteries, independently of hypertension and other vascular factors, has an important impact on cognitive function in the aging population.^4–6^ Hence, these studies stress the need to better understand how arterial stiffness affects brain homeostasis in order to establish preventive interventions and treatments that will protect cognitive function in the elderly.

Although it is now well recognized that arterial stiffness could lead to end‐organ damage, there is currently no animal model to study its effects on the brain. The existing animal models of arterial stiffness fall into 2 main categories. The first category includes animal models in which arterial stiffening is secondary to another condition such as aging and atherosclerosis, whereas the second one encompasses animals in which arterial stiffness is induced by chemical, surgical, or genetic means.^7^ However, in all these models the proposed interventions can parallelly affect the brain. For example, the frequently used vitamin D/nicotine and vitamin K/warfarin models achieve arterial stiffness by arterial calcification.^8–10^ It is believed that vascular calcification directly induces arterial stiffness, as has been shown in these models as well as in hypertensive and diabetic patients.^7^ However, compounds such as vitamins D and K are known to directly interact with the central nervous system.^11–12^ The surgical models of induction of arterial stiffness, such as ischemia and the replacement of an artery by a stiff tube, substantially decrease cerebral blood flow during the surgery, which may thus affect the brain.^13–14^ Finally, the genetic modifications specific to the extracellular matrix (elastin or fibrillin) or the ones inducing general arterial calcification are not specific to blood vessels or to a precise segment of the arterial tree, therefore simultaneously affecting many organ functions.^15^ Although all these animal models can be used to study arterial stiffness, they all lack an important feature, the specificity for arterial stiffness. Moreover, arterial stiffness can also be accompanied by other confounding variables such as physiological changes induced by systemic high blood pressure, vessel stenosis leading to cerebral hypoperfusion, and high levels of circulating lipids as well as heart calcification and bradycardia.^7,9,16–19^ Therefore, there is a need to develop a new model that would allow the study of the precise outcome of arterial stiffness on the brain's health.

Hence, we have developed a new murine model of arterial stiffness that has been adapted from an existing model of elastocalcinosis and aneurysm in which a periadventitial application of calcium chloride (CaCl_2_) to the carotid artery or aorta induces calcification of elastic fibers.^20–23^ This is the first model allowing the study of the specific role of arterial stiffness on brain structure and homeostasis. Here, we show that periarterial application of CaCl_2_ to carotid arteries induces arterial stiffness, as shown by decreased compliance of calcified arteries, enhanced collagen distribution, fragmentation of elastin, and increased intima–media thickness. Regarding the brain, this model exhibits an increase in cerebral blood flow pulsatility, increased superoxide anion production, and neurodegeneration. Therefore, we demonstrate here that this new model of arterial stiffness can be used to assess the specific effects of carotid stiffening on the brain's health.

## Methods

### Animals

Ten‐ to 12‐week‐old male C57BL/6 mice were purchased from Charles River Laboratories (Saint‐Constant, Quebec, Canada) and housed individually in a temperature‐controlled room with 12‐hour light–dark cycles. Following acclimatization, the mice were divided into groups of 3 to 12 mice each before being treated for 2 weeks. The study was approved by the Animal Care and Use Committee of the Université de Montréal and performed in accordance with the guidelines of the Canadian Council for Animal Care.

### Periarterial Application of CaCl_2_

Anesthesia was induced by placing mice in a closed chamber containing 5% isoflurane and 3 L/min of oxygen and maintained with a mixture of 1.5% to 2% isoflurane and 1.5 to 2 L/min of oxygen. Throughout the surgery, the level of anesthesia was monitored by testing corneal reflexes and motor responses to tail pinch. Each animal's temperature was maintained using a heating pad, and the procedure was performed in sterile conditions. The incision site was sanitized with a solution of povidone‐iodine and 70% isopropyl alcohol. An incision of ≈1.5 cm was made, and the trachea was exposed by carefully separating the submandibular glands with sterile cotton swabs. Tissue hydration was maintained throughout the procedure with a sterile saline solution. The right common carotid artery was carefully isolated by sliding a small piece of sterile parafilm underneath it. Sterile cotton gauze soaked in 0.2, 0.3, and 0.4 mol/L CaCl_2_ or 0.9% NaCl (control) was placed directly on the carotid artery for 20 minutes. The gauze was then removed, and the incision was closed using 6‐0 silk sutures and Vetbond tissue glue. The entire procedure lasted 40 minutes. The discomfort caused by the incision was prevented by the administration of bupivacaine hydrochloride (Marcaine; 4 mg/kg subcutaneous injection at the site of the incision) and carprofen (Rimadyl; 5 mg/kg subcutaneous injection) immediately after surgery. In addition, carprofen (5 mg/kg subcutaneous injection) was administered every 24 hours for 2 days following surgery. Infections were prevented by the administration of trimethoprim sulfadiazine (Tribrissen; 30 mg/kg subcutaneous injection) immediately after surgery and every 24 hours for 3 days. The calcification of both carotid arteries at the same time was tested but discontinued because of the high rate of mortality.

### Carotid Artery Histological Assessment

Carotid arteries were embedded in paraffin, cut on a microtome (10 μm), and processed for Von Kossa and Masson's Trichrome stains (histology core facility of the Institute for Research in Immunology and Cancer, Université de Montréal, Montreal, Quebec, Canada, histological protocols are described in details in the Supplemental material section). Images were acquired with a Leica DM200 microscope (40× magnification). Carotid intima–media thickness was measured in arteries stained with Masson's Trichrome. Carotid sections were deparaffinized and used to detect the presence of macrophages by immunofluorescence using rat anti‐mouse macrophage/monocyte antibody (MOMA‐2) coupled with Alexa Fluor 647 (AbD Serotec).^24–25^ To assure uniformity of the immunolabel, sections from all groups were processed together. Elastin distribution was determined by autofluorescence using an Olympus laser scanning confocal microscope (488 nm excitation/550 to 600 nm emission). Image acquisitions were performed with the same fluorescence settings in all cases with a computer‐controlled digital camera. Data are expressed in arbitrary fluorescence units.

### Carotid Compliance Studies

Compliance was measured in passive conditions to reflect the mechanical properties of the vascular wall. Carotid arteries were mounted on a pressure myograph (Living Systems Instrumentation) and pressurized at 60 mm Hg. Diameter changes were measured by video microscopy from 60 to 180 mm Hg with steps of 20 mm Hg in a Ca^2+^‐free physiological salt solution (pH 7.4; 130 mmol/L NaCl, 4.7 mmol/L KCl, 1.18 mmol/L KH_2_PO_4_, 1.17 mmol/L MgSO_4_, 14.9 mmol/L NaHCO_3_, 0.023 mmol/L EDTA, and 10 mmol/L glucose) containing 1 mmol/L EGTA to abolish myogenic tone and to uniquely assess the mechanical properties of the arteries.^19,26^ The protocol is described in details in the Supplemental material section. The initial diameter of 60 mm Hg was noted to assess the carotid's radius. The circumferential wall strain (%) was determined by (D−D_initial mm Hg_)/D_initial mm Hg_), where D is the diameter at a given pressure and D_initial mm Hg_ is the initial diameter at 60 mm Hg. The incremental distensibility (%/mm Hg) was determined by (D_1_−D_0_)/(D_1_×ΔP)×100, where D_0_ and D_1_ are the internal diameters before and after pressure increment, respectively, and ΔP is the change in pressure (20 mm Hg).^19^ The β index of stiffness was determined by β=ln(Ps/Pd)/(Ds−Dd)/Dd, where Ps and Pd are the in vivo systolic and diastolic pressures and Ds and Dd are the carotid diameters corresponding to these pressures.^27^

### Flow Pulsatility Studies

Doppler optical coherence tomography (OCT) was used to measure blood flow changes in different areas of the brain. Two weeks following periarterial application of 0.3 mol/L CaCl_2_ or 0.9% NaCl (control), the mice were anesthetized with urethane 10% weight/volume (200 μL/10 g) and placed in a stereotaxic stage (Harvard Apparatus). A total of 22 mice were studied, 11 for each group. Mouse body temperature was maintained using a heating pad (MouseSTAT, Kent Scientific), and heart rate and temperature were carefully monitored throughout the experience. Following removal of the skin, the brain was exposed, and the imaging was performed over the frontal and temporal lobes. For each mouse, between 4 and 6 vessels were imaged under the OCT system. For each artery, 2 perpendicular B‐mode scans were performed, and the blood speed profile was obtained during a single cardiac cycle. Two pulsatility metrics were obtained from the cardiac cycle profile of each slice: first, the relative blood speed increase from the minimum speed to the maximum; second, the blood speed variability, defined as the standard deviation divided by the mean. Vessel diameter was estimated as the smallest cross section of the vessel measured in both perpendicular scans.^28^ The protocol and analysis approach are described in details in the Supplemental material section.

### Blood Pressure Assessment

Blood pressure was monitored by a noninvasive volumetric tail‐cuff method (Coda Kent Scientific Corporation) every 4 days from the day of surgery until the end of the 2 weeks of treatment. Mice were placed on a heating platform for 10 to 15 minutes before assessment of blood pressure. A minimum of 5 measurements were taken until the blood pressure stabilized, and a minimum of 10 measurements were taken per mouse. The measurements were taken at the same time of the day.

### Dihydroethidium Staining

Frozen brains were cut on a cryostat (20 μm), and sections were stained with fluorescent‐labeled dihydroethidium (DHE) solution (2 μmol/L; Sigma‐Aldrich). Images were acquired using an epifluorescence microscope Leica DM200 (40× magnification) with the same fluorescence settings. Analysis of relative fluorescence was achieved using Image J software (version 1.47; National Institutes of Health). The protocol is described in details in the Supplemental material section.

### Fluoro‐Jade B Staining

The method was adapted from Schmued et al.^29^ Briefly, frozen brain sections were immersed in 0.06% potassium permanganate followed by Fluoro‐Jade B 0.0008% solution (Millipore) in 0.1% acetic acid vehicle. Images were acquired with an epifluorescence microscope Leica DM200 (40× magnification) with the same fluorescence settings. Analysis of percentage of the total area occupied by Fluoro‐Jade B–positive cells was achieved using Image J software (version 1.47; National Institutes of Health). The protocol is described in details in the Supplemental material section.

### Statistical Analysis

Results are presented as mean±standard error of the mean. Multiple comparisons were accounted for by 1‐way analysis of variance with Bonferroni post hoc analysis, and 2‐group comparisons of independent samples were analyzed by an unpaired 2‐tailed Student *t* test. Blood speed increases from the left and right sides of the brain were compared using a paired 2‐tailed Student *t* test. Analysis was achieved using GraphPad Prism 5.01. Statistical significance was set at *P*<0.05.

## Results

### Periarterial Application of CaCl_2_ to Carotid Arteries Modifies Their Composition

**Figure 1.**
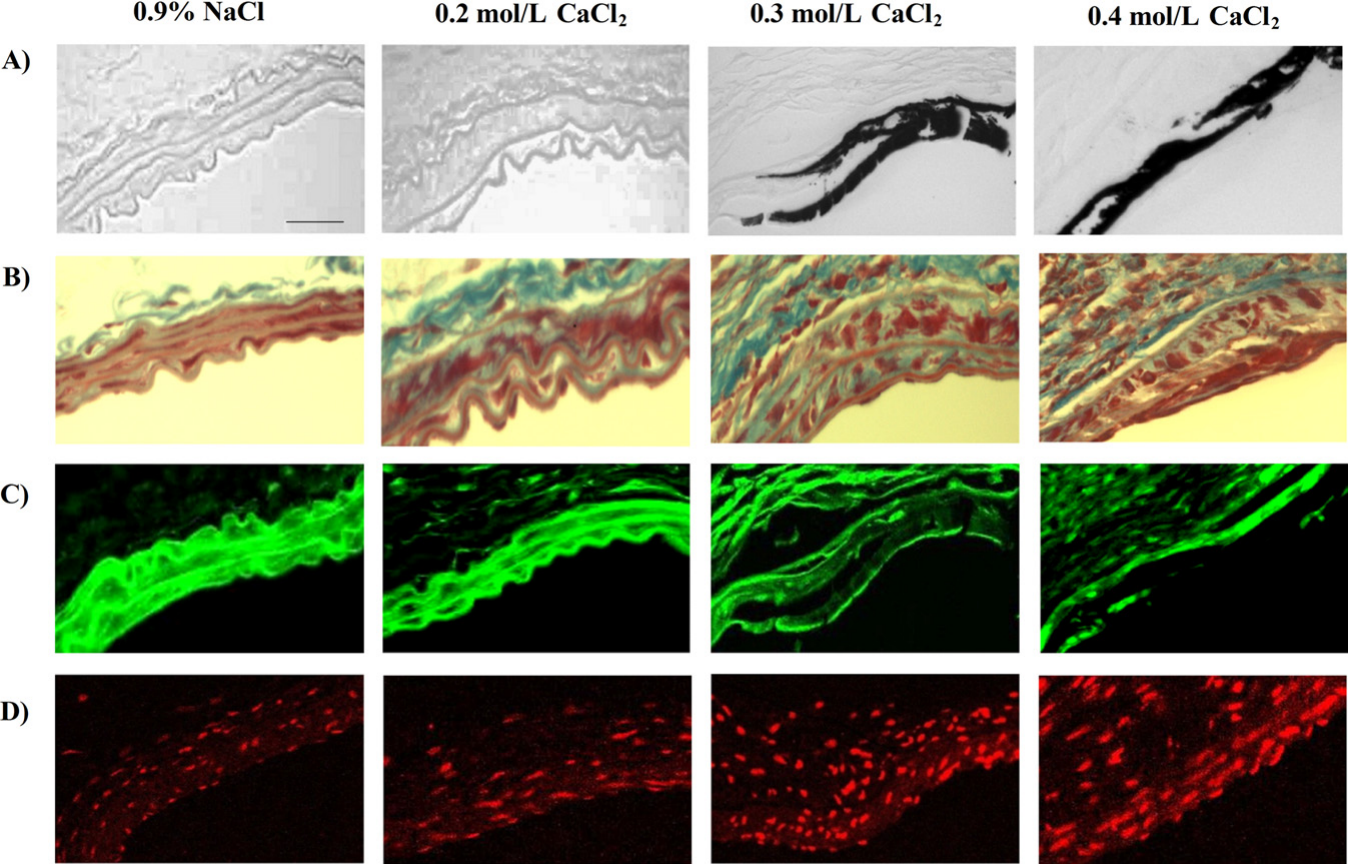
Calcium deposits, collagen and elastin distribution, and macrophage infiltration in carotid arteries submitted to periarterial application of CaCl_2_. Representative sections of carotid arteries 2 weeks following application of CaCl_2_ or 0.9% NaCl showing calcium deposits labeled in black with Von Kossa stain (A), collagen deposits labeled in blue with Masson's Trichrome stain (B), elastin distribution labeled in green by autofluorescence (C), and macrophage/monocyte infiltration labeled in red using MOMA‐2 (1/50) antibody (D) (n=6, scale=25 μm). CaCl_2_ indicates calcium chloride; NaCl, sodium chloride; MOMA, macrophage/monocyte antibody.

To assess the efficiency of periarterial application of CaCl_2_ to induce calcium incorporation into the vasculature, the presence of calcium deposits was confirmed by Von Kossa stain. As shown in Figure 1, periarterial application of 0.3 and 0.4 mol/L CaCl_2_ induces formation of calcium deposits (black spots in the carotid tissue) compared with the control 0.9% NaCl, with which the tissue remains intact (Figure 1A). These calcium deposits were observed only in the carotid artery area where the calcium‐soaked gauze was applied (data not shown). To assess histological characteristics of arterial stiffness, collagen and elastin distribution were evaluated. Application of CaCl_2_ increased collagen deposits for all the CaCl_2_ doses but more considerably for the higher doses of 0.3 and 0.4 mol/L (Figure 1B). As for elastin, visualized by autofluorescence, a fragmentation of elastin was observed for 0.3 and 0.4 mol/L of CaCl_2_ (Figure 1C). Finally, periarterial application of CaCl_2_ induced infiltration of macrophages (Figure 1D), indicating the presence of inflammation at the sites where CaCl_2_ was applied. These effects were observed 2 weeks following carotid calcification. This point was chosen because 1 week following CaCl_2_ application, no difference in collagen or elastin distribution was observed compared with controls, and after 3 weeks, calcium deposits and collagen increases tended to resorb (data not shown).

### Periarterial Application of CaCl_2_ Increases Intima–Media Thickness of Carotid Arteries Without Affecting Its Radius

**Figure 2.**
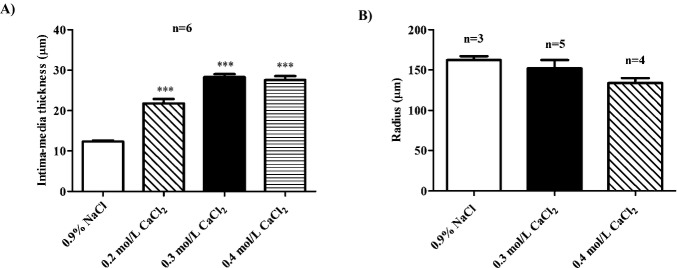
Geometric characteristics of carotid arteries exposed to periarterial application of CaCl_2_. Measurement of intima–media thickness (A) and radius (B) in carotid arteries 2 weeks following periarterial application of different concentrations of CaCl_2_ or 0.9% NaCl (control) (n=3 to 6; ***P<0.001 vs 0.9% NaCl). CaCl_2_ indicates calcium chloride; NaCl, sodium chloride.

To evaluate the geometric properties of arterial stiffness, the carotid radius and its intima–media thickness were measured. CaCl_2_ application induced a significant increase in intima–media thickness for all doses of CaCl_2_, with higher increases observed for the higher doses of 0.3 and 0.4 mol/L CaCl_2_ (*P*<0.001; Figure 2A)_._ Indeed, the carotid artery intima–media thickness exposed to NaCl was 12.3±0.3 μm, and it increased to 21.8±1.1, 28.3±0.7, and 27.5±1.0 μm in the carotid arteries exposed to 0.2, 0.3, and 0.4 mol/L of CaCl_2_, respectively. However, the arterial radius was not altered by CaCl_2_ application (Figure 2B). The radius of carotids exposed to NaCl was 162.2±4.7 μm, whereas those subjected to periarterial application of CaCl_2_ did not show any increase in arterial radius (the radius was equal to 152.0±10.3 μm in carotids subjected to 0.3 mol/L CaCl_2_ and to 133.8±6.2 μm in carotids exposed to 0.4 mol/L CaCl_2_).

### Carotid Calcification Decreases Carotid Compliance and Distensibility

**Figure 3.**
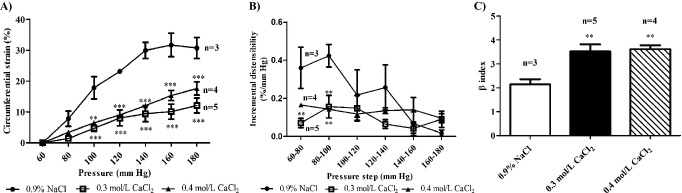
Effect of periarterial application of CaCl_2_ on carotid compliance, distensibility, and β index. Measurement of carotid compliance, expressed as circumferential strain (A), incremental distensibility (B), and β stiffness index (C), 2 weeks following periarterial application of different concentrations of CaCl_2_ or 0.9% NaCl (control) (n=3 to 5; ***P<0.001 vs 0.9% NaCl, **P<0.01 vs 0.9% NaCl). CaCl_2_ indicates calcium chloride; NaCl, sodium chloride.

To evaluate the mechanical properties of arterial stiffness, carotid compliance and distensibility were determined. Compared with controls (0.9% NaCl), carotid arteries subjected to periarterial application of 0.3 and 0.4 mol/L CaCl_2_ were less compliant, as illustrated by a decrease in circumferential strain for pressures varying from 100 to 180 mm Hg (*P*<0.01; Figure 3A). Moreover, carotid calcification induced a significant decrease in incremental distensibility of between 60 and 100 mm Hg for the doses of 0.3 and 0.4 mol/L (*P*<0.01; Figure 3B). Finally, periarterial application of 0.3 and 0.4 mol/L CaCl_2_ induced an important and significant increase in the β index, an index of arterial stiffness, compared with controls (0.9% NaCl; *P*<0.01; Figure 3C). Indeed, the β index was 2.2±0.2 for carotids exposed to NaCl, and it increased to 3.5±0.3 and to 3.6±0.2 for carotids exposed to 0.3 and 0.4 mol/L CaCl_2_, respectively (*P*<0.01). Overall, these data indicate an increase in carotid stiffness induced by carotid calcification.

### Carotid Calcification Increases Cerebral Blood Flow Pulsatility

**Figure 4.**
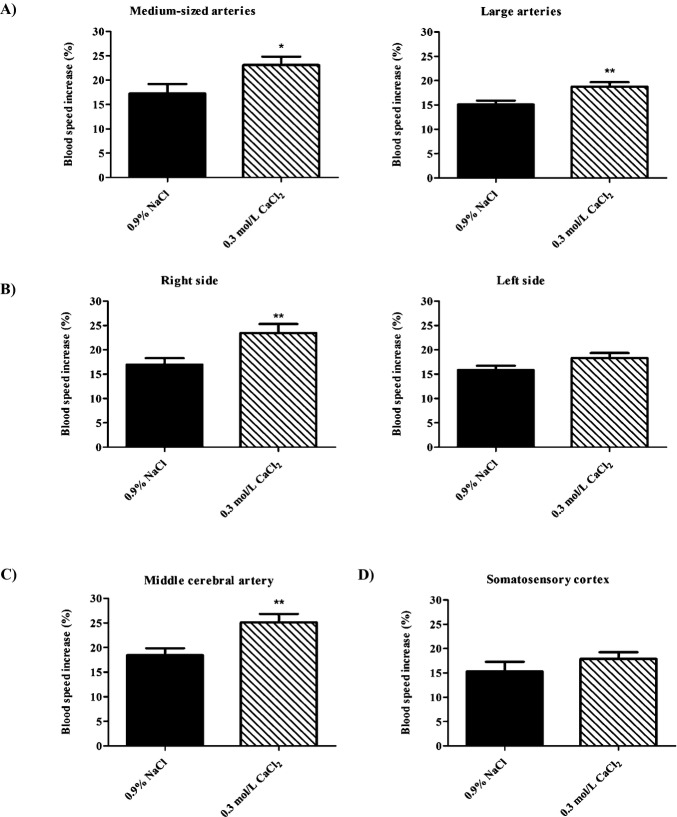
Effect of periarterial application of 0.3 mol/L CaCl_2_ on cerebral blood flow pulsatility. Blood flow pulsatility, represented as blood speed increase (%) in medium‐sized arteries with a diameter varying from 50 to 95 μm and in large arteries with a diameter >95 μm (A), in the right side of the brain corresponding to the right common carotid artery that was calcified and the left side of the brain corresponding to the intact carotid (B) as well as at the level of the middle cerebral artery and its branches in the parietal cortex (C) and in the arteries of the somatosensory cortex (D) (n=11; **P<0.01 vs 0.9% NaCl, *P<0.05 vs 0.9% NaCl). CaCl_2_ indicates calcium chloride; NaCl, sodium chloride.

Upstream blood flow pulsatility, also an important component of stiffness in large arteries, was assessed in the brain by measuring blood speed increases in different arterial segments. On the basis of histological and mechanical assessment of arterial stiffness, the dose of 0.3 mol/L CaCl_2_ was chosen as an optimal dose to induce carotid artery stiffness. For analysis, vessels were divided into 2 groups: medium‐sized arteries with a diameter <95 μm and large arteries with a diameter >95 μm. Hence, carotid stiffness induced by its calcification significantly increased blood flow pulsatility in upstream medium‐sized arteries with a diameter varying from 50 to 95 μm (*P*=0.041; Figure 4A) and in large arteries with a diameter >95 μm (*P*<0.01; Figure 4A). In medium‐sized arteries, the percentage of blood speed increase changed from 17.2±5.2% for the control group (0.9% NaCl) to 23.1±5.1% for the CaCl_2_ group. In large arteries, blood speed increase evolved from 15.1±0.8% for the control to 18.7±1.0% for the CaCl_2_ group (*P*<0.01). Moreover, the right side of the brain corresponding to the right common carotid artery that was calcified showed a significant increase in blood speed, shifting from 16.9±4.3% for control group to 23.5±5.7% for the CaCl_2_ group (Figure 4B, *P*<0.01). The left side of the brain corresponding to the intact carotid artery showed a nonsignificant increase in blood speed (Figure 4B). Regarding the specific regions of the brain, carotid calcification induced a significant increase in blood speed in the middle cerebral artery and its branches, measured in the parietal cortex (*P*<0.01; Figure 4C), whereas there was no significant increase in blood speed observed in the vessels of the somatosensory cortex (Figure 4D). Indeed, for the middle cerebral artery and its branches, blood speed increase evolved from 18.5±3.9% for the control group to 25.1±4.8% for the CaCl_2_ group. In the somatosensory cortex, the control group showed a blood speed increase of 15.3±6.2%, whereas the CaCl_2_ group showed an increase of 17.9±4.0%. These data demonstrate that the carotid stiffness‐induced increase in cerebral blood flow pulsatility differs according to the different arterial segments, probably depending on the distance.

### Blood Pressure Assessment

To alleviate the possibility that the effects of carotid stiffness observed in the brain were related to increased systemic blood pressure and its associated physiological responses, systolic blood pressure was measured every 4 days from the day of surgery until the end of the treatment (Table1). As shown, before surgery (day 0), the pressure was similar between the controls (0.9% NaCl) and the different groups of CaCl_2_. The blood pressure remained stable without any increase during the whole treatment period (14 days), indicating that CaCl_2_ does not alter blood pressure.

**Table 1.** Blood Pressure Assessment

	0.9% NaCl	0.3 mol/L CaCl_2_	0.4 mol/L CaCl_2_
Day 0	135.2±4.5	129.6±3.1	134.9±4.0
Day 4	133.3±3.6	135.1±3.2	136.8±2.5
Day 8	127.3±2.9	129.5±3.3	131.9±3.4
Day 14	130.2±2.3	127.4±2.4	129.9±1.9

Systolic blood pressure (mm Hg) was monitored on days 4, 8, and 14 from the day of surgery (day 0) until the end of the treatment (2 weeks) by noninvasive volumetric tail‐cuff measurement (n=6). NaCl indicates sodium chloride; CaCl_2_, calcium chloride.

### Carotid Stiffness, Induced by Its Calcification, Increases Superoxide Anion Production in the Hippocampus

**Figure 5.**
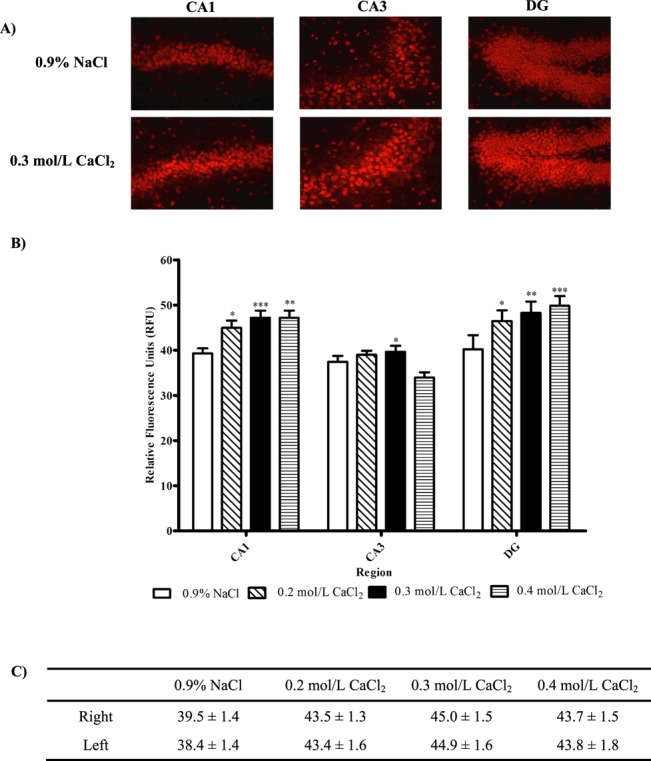
Effect of carotid stiffness, induced by periarterial application of CaCl_2_, on oxidative stress, assessed by superoxide anion production in the hippocampus. A, Representative sections of different regions of the hippocampus (×40 magnification), stained with DHE, in mice with carotid arteries submitted to application of 0.3 mol/L CaCl_2_ or 0.9% NaCl (control). B, Superoxide anion production assessed by DHE fluorescence (relative fluorescence units) in CA1, CA3, and DG regions of hippocampus for different concentrations of CaCl_2_ or 0.9% NaCl (control). C, Superoxide anion production assessed by DHE fluorescence (relative fluorescence units) in right or left hemisphere of the hippocampus for different concentrations of CaCl_2_ or 0.9% NaCl (n=3; ***P<0.001 vs 0.9% NaCl, **P<0.01 vs 0.9% NaCl, *P<0.05 vs 0.9% NaCl). CaCl_2_ indicates calcium chloride; NaCl, sodium chloride; DHE, dihydroethidium; CA1 and CA3, cornu ammonis 1 and 3; DG, dentate gyrus.

Next, we sought to determine whether arterial stiffness alters brain homeostasis. Therefore, superoxide anion levels were quantified to evaluate the degree of oxidative stress present in the brain. As shown, carotid stiffness induced a significant 1.2‐fold increase in superoxide anion production, a reactive oxygen species, for the dose of 0.3 mol/L CaCl_2_ in all regions of the hippocampus, cornu ammonis 1 and 3 (CA1 and CA3), and dentate gyrus (DG) (*P*<0.01; Figure 5A). An increase in superoxide anion levels was also noted for the doses of 0.2 mol/L, a 1.1‐fold increase, and 0.4 mol/L, a 1.2‐fold increase, in the CA1 and DG regions of the hippocampus (*P*<0.01; Figure 5B). The left and right sides of the brain were compared for superoxide anion levels, and no side‐to‐side difference was observed (Figure 5C). Regarding the cortical areas of the brain, such as the frontal and somatosensory cortices, no increase in superoxide anion production was found (data not shown).

### Fluoro‐Jade B Staining Reveals Presence of Neurodegeneration

**Figure 6.**
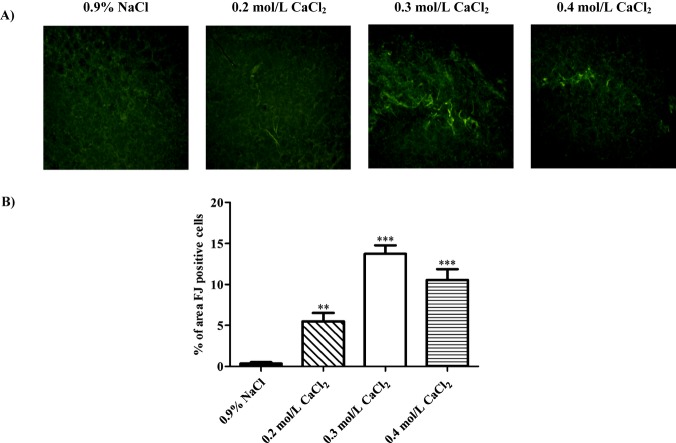
Presence of neurodegeneration in hippocampus of mice with carotid stiffness. Representative hippocampal sections, stained with Fluoro‐Jade (FJ) B (A) and percentage of area occupied by FJ B–positive cells (B) 2 weeks following periarterial application of different CaCl_2_ concentrations or 0.9% NaCl (control) (n=5). CaCl_2_ indicates calcium chloride; NaCl, sodium chloride.

The increased oxidative stress in the hippocampus suggests that neurons might also be affected. The presence of degenerative neurons was assessed with Fluoro‐Jade B stain. Periarterial application of CaCl_2_ induced neurodegeneration in the CA1 region of the hippocampus, as shown by increases in the percentage of the area occupied by Fluoro‐Jade B–positive cells for the doses of 0.3 and 0.4 mol/L CaCl_2_ compared with controls (*P*<0.01; Figure 6). Indeed, the percentage of area occupied by degenerative neurons evolved from 0.4±0.2% for the control group to 5.5±1.1%, 13.7±1.0%, and 10.6±1.3% for the groups exposed to 0.2, 0.3, and 0.4 mol/L CaCl_2_, respectively. No difference was observed between the sides of the brain, left or right hemisphere (data not shown). The presence of degenerative neurons is specific to a subarea of the CA1 region called lacunosum moleculare. No neurodegeneration was found in the cortex or in other regions of the hippocampus, such as DG and CA3 (data not shown).

## Discussion

Studying the effect of arterial stiffness on the brain's health has been difficult because of the lack of good animal models. In the present study, we developed a new model that meets all the characteristics of arterial stiffness in large arteries without any unspecific effects such as global brain hypoperfusion or increased systemic blood pressure. This new model, induced by application of CaCl_2_ to the carotid arteries of mice, displays increased arterial collagen deposition and intima–media thickness as well as elastin fragmentation leading to decreased arterial compliance. This model also exhibits altered brain homeostasis. Indeed, the study of the cerebral effects shows increased cerebral blood flow pulsatility and increased oxidative stress accompanied by neuronal damage.

Recently, arterial stiffness has been shown to be an important predictor of cognitive decline in the elderly, as well as an independent predictor of stroke.^1–6^ Therefore, its effect on brain homeostasis needs to be further explored. To allow this kind of study, an efficient model of arterial stiffness needed to be developed to study the precise outcome of arterial stiffness in the brain without any interaction with other cardiovascular factors. Hence, we developed a new model of arterial stiffness based on the calcification of carotid arteries in mice. Carotid arteries were chosen because of their proximity to the brain and of studies that have shown that carotid atherosclerosis is an important predictor of stroke and that the role of carotid stiffness in cerebrovascular function needs to be further examined.^7,30^

The new model was characterized on the basis of different characteristics of arterial stiffness. First, the efficiency of the periarterial application of CaCl_2_ to carotid arteries was confirmed by calcium deposits at higher doses of CaCl_2_. It is important to note that these calcium deposits were only present at the site of the application of the calcium‐soaked gauze. Therefore, the calcification of carotid arteries did not affect the entire arterial segment and was concentrated at the site of carotid injury, opposite to other models such as the vitamin D/nicotine model, which lacks specificity for a particular arterial segment.^8^ The anatomical characteristics of arterial stiffness were confirmed by increased collagen deposition, especially for the doses of 0.3 and 0.4 mol/L CaCl_2_. Collagen increases wall thickness, which contributes greatly to arterial stiffness.^7,31^ Elastin may be fragmented or its content reduced in stiff arteries. Moreover, it has been shown that direct calcium deposition on arteries induces elastin fragmentation at the site of calcium deposits.^21^ Indeed, in our model, elastin fragmentation can be seen at the sites of calcium deposits for the higher doses of CaCl_2_. Elastin distribution remains intact for the rest of the artery wall where calcium deposits are absent. Moreover, the periarterial application of CaCl_2_ induces an increase in macrophage infiltration, leading to local inflammation, also a component of arterial stiffness.^32^ Two weeks was chosen as an optimal follow‐up time, as it is at that period that calcium deposits were clearly distinct and collagen and elastin distribution were significantly altered. At 3 weeks, these effects began to resorb. This is a limitation in this model for the study of more chronic effects on the brain.

To further characterize arterial stiffness, the geometry of carotid arteries was evaluated. Carotid calcification increases the intima–media thickness without affecting its radius. It has been shown that common carotid intima–media thickness is strongly correlated with the risk of stroke.^7^ Therefore, this model encompasses different components of arterial stiffness related to increased risk of cerebrovascular events. As the carotid artery radius remains unchanged, this indicates that the doses of CaCl_2_ used do not induce aneurysm, as in previous models of periadventitial application of higher doses of CaCl_2_,^20,23^ and that the effects observed in the brain are due particularly to the induced stiffness rather than global brain hypoperfusion. However, it does not exclude the possibility that cerebral blood flow is altered at the level of arterioles or capillaries. Hence, an assessment of cerebral blood flow would be interesting to examine this possibility.

To further confirm that the carotid arteries are stiff, its mechanical properties were assessed. Carotid arteries showed decreased arterial compliance, indicating that the calcified arteries do not dilate as much as controls, especially at higher pressures (100 to 180 mm Hg). The calcified carotids also showed decreased distensibility, which represents the relative diameter change for a pressure increment. Hence, these data confirm that periarterial application of CaCl_2_ induces carotid stiffness. Moreover, carotid stiffness increases the β index, which is a common clinical marker used to assess arterial mechanical properties and is considered, with intima–media thickness measurement, an early marker and predictor of atherosclerosis.^33^ Moreover, Jurasic et al conducted a study to measure the β stiffness index in young and elderly populations with no serious cardiovascular conditions. The study showed that the elderly population (65 to 75 years) had a β index ≈1.6 times higher than the young population (25 to 35 years). In comparing this to mice, the control group of adult mice (0.9% NaCl) corresponds to the young population. As the data showed, the group of mice with calcified and stiffened arteries (0.3 and 0.4 mol/L CaCl_2_) had a β index ≈1.6 times higher than that of the control group. This indicates that, based on the β stiffness index, this new animal model of arterial stiffness may clinically relate to a population of 65 to 75 years old. Therefore, on the basis of histological and mechanical assessment of arterial stiffness, the dose of 0.3 mol/L CaCl_2_ was chosen as the optimal dose to induce arterial calcification leading to its stiffness. Arterial stiffness can also be influenced by functional components such as smooth muscle reactivity mediated by nitric oxide and sympathetic innervation. However, vasoconstrictor tone exerted by smooth muscle cells as well as endothelial function is much less important in a conduit artery such as the carotid.^34^ Indeed, in conduit arteries, structural factors dominate over functional ones in determining arterial stiffness, because the smaller amount and different arrangement of smooth muscle in the wall may not exert significant influence on vessel diameter and distending pressure. In addition, arterial stiffness can often be accompanied by a narrowing of the lumen caused by vascular remodeling or atherosclerotic plaques and also by the effects on systolic blood pressure. However, many studies have also demonstrated that some individuals can present high arterial stiffness that is not accompanied by systolic hypertension.^35–37^ Hence, the global clinical application of this study is to find a parameter that occurs before increases in blood pressure and that can be acted on to reduce the risk or to prevent the effects on the brain.^38^

A third important component of arterial stiffness is increased blood flow pulsatility. More specifically, large elastic arteries, such as the carotid and aorta, regulate pulsatile flow to maintain the integrity of the microvasculature. When these arteries become stiff, their capacity to damper pulsatile flow is reduced. There is growing evidence that increased large artery stiffness induces excessive flow pulsatility that contributes to dysfunction, especially in the brain and kidneys, which are high–blood flow organs and which are more sensitive to excessive flow pulsatility.^39–41^ Our data show that carotid stiffness induces an increase in blood flow pulsatility in the brain; it affects medium‐ and large‐sized arterial segments. Moreover, the side of the brain corresponding to the carotid artery that was calcified shows a more important increase in flow pulsatility as opposed to the side of the brain corresponding to the intact carotid. These results are compatible with the principle that flow pulsatility decreases with distance from the central arteries.^42^ Heart rate was monitored during the procedure and did not differ between the control group and the group with carotid calcification. Blood pressure was assessed to confirm that the increased blood flow pulsatility seen in the brain is not a result of increased systemic blood pressure. As the data show, blood pressure remained constant among the controls and mice that received CaCl_2_ application, indicating that carotid stiffness is the only factor contributing to increased flow pulsatility in the brain. This also adds to the strength of this new model; carotid calcification induces arterial stiffness without affecting systemic blood pressure, which can trigger many neuronal and hormonal responses, therefore isolating arterial stiffness in order to study its effects on the brain.

Previous studies have shown that blood flow pulsatility induces an increase in oxidative stress, which is the first step of potential damage.^43–44^ Therefore, superoxide anion levels were determined in different brain regions. The data show that carotid stiffness induces a significant increase in the production of superoxide anions in all regions of the hippocampus. The presence of increased oxidative stress in the hippocampus suggests that brain homeostasis is disturbed and that neurons might be affected. To assess more precisely the potential neuronal damage, the presence of neurodegeneration was determined. Indeed, carotid calcification induces neurodegeneration in the hippocampus. Interestingly, the presence of degenerative neurons is specific to a subarea of the CA1 region called lacunosum moleculare. This region contains interneurons that integrate signals between the entorhinal cortex and the CA1 region of the hippocampus and that are important for episodic and spatial memory. It has been shown that entorhinal neurons that project to the hippocampus are among the first cells that are affected in Alzheimer's disease.^45^ However, the effect on oxidative stress and neurons is absent in the frontal and somatosensory cortices. These results are also compatible with the observation that pulsatility decreases with distance from the central vessels^42^ and could also explain why no difference in oxidative stress was observed between the right and left sides of the hippocampus. Indeed, although the increased pulsatility induced by carotid calcification was generally lower in the left hemisphere, this was measured in the cortex, and as the pulsatility decreases with the distance, this difference between the 2 sides is probably much less significant in regions close to the large arteries of the brain. Moreover, it has been shown that changes in cerebral arterial properties, such as the ones occurring during arteriosclerosis, lead mainly to damage in vessels closer to the main arteries that perfuse the brain. Hence, the central regions of the brain are more affected by these changes,^46^ thus making regions such as the hippocampus and amygdala more vulnerable to damage compared with regions at a greater distance such as the cortex. Indeed, a strong correlation between regions of high pulsatile stress and decreased hippocampal volume has been observed.^42^ These results are also compatible with those in Alzheimer's disease patients in whom the spatial distribution of gray matter loss correlates with regions of high perfusion rates and proximity of large arteries.^47^ Moreover, because of their high metabolism, hippocampal cells, especially in the CA1 region, are considered a more vulnerable cell population in response to vascular dysfunctions, making it more prone to damage induced by carotid calcification.^48^ Overall, these data indicate that arterial stiffness, induced by carotid calcification, affects the brain and that this new model should be used to further study this aspect.

In conclusion, we have developed a new model of arterial stiffness based on the calcification of carotid arteries in mice. This model exhibits the essential characteristics of arterial stiffness in large arteries, which are: (1) an increase in collagen distribution and in intima–media thickness as well as elastin fragmentation, (2) a decrease in arterial compliance and distensibility, and (3) an increase in cerebral blood flow pulsatility. Regarding the brain, carotid stiffness induces an increase in oxidative stress and neurodegeneration in the hippocampus. This new model indicates that arterial stiffness may play an important role in the pathogenesis of neurodegenerative diseases and that this new model may be used to study the precise outcome of arterial stiffness on the brain's health.
